# Transcriptional time course after rotator cuff repair in 6 month old female rabbits

**DOI:** 10.3389/fphys.2023.1164055

**Published:** 2023-05-09

**Authors:** Laura S. Vasquez-Bolanos, Michael C. Gibbons, Severin Ruoss, Isabella T. Wu, Mary C. Esparza, Donald C. Fithian, John G. Lane, Anshuman Singh, Chanond A. Nasamran, Kathleen M. Fisch, Samuel R. Ward

**Affiliations:** ^1^ Department of Bioengineering, University of California, San Diego, San Diego, CA, United States; ^2^ Department of Orthopaedic Surgery, University of California, San Diego, San Diego, CA, United States; ^3^ Department of Orthopaedic Surgery, Kaiser Permanente, San Diego, CA, United States; ^4^ Center for Computational Biology and Bioinformatics, Department of Medicine, University of California, San Diego, San Diego, CA, United States; ^5^ Department of Obstetrics, Gynecology and Reproductive Science, University of California, San Diego, San Diego, CA, United States; ^6^ Department of Radiology, University of California, San Diego, San Diego, CA, United States

**Keywords:** rotator cuff repair, transcriptome analysis, time series data, rotator cuff muscle dysfunction, muscle biology, muscle atrophy

## Abstract

**Introduction:** Rotator cuff tears are prevalent in the population above the age of 60. The disease progression leads to muscle atrophy, fibrosis, and fatty infiltration, which is not improved upon with surgical repair, highlighting the need to better understand the underlying biology impairing more favorable outcomes.

**Methods:** In this study, we collected supraspinatus muscle tissue from 6 month old female rabbits who had undergone unilateral tenotomy for 8 weeks at 1, 2, 4, or 8 weeks post-repair (*n* = 4/group). RNA sequencing and enrichment analyses were performed to identify a transcriptional timeline of rotator cuff muscle adaptations and related morphological sequelae.

**Results:** There were differentially expressed (DE) genes at 1 (819 up/210 down), 2 (776/120), and 4 (63/27) weeks post-repair, with none at 8 week post-repair. Of the time points with DE genes, there were 1092 unique DE genes and 442 shared genes, highlighting that there are changing processes in the muscle at each time point. Broadly, 1-week post-repair differentially expressed genes were significantly enriched in pathways of metabolism and energetic activity, binding, and regulation. Many were also significantly enriched at 2 weeks, with the addition of NIF/NF-kappaB signaling, transcription in response to hypoxia, and mRNA stability alongside many additional pathways. There was also a shift in transcriptional activity at 4 weeks post-repair with significantly enriched pathways for lipids, hormones, apoptosis, and cytokine activity, despite an overall decrease in the number of differentially expressed genes. At 8 weeks post-repair there were no DE genes when compared to control. These transcriptional profiles were correlated with the histological findings of increased fat, degeneration, and fibrosis. Specifically, correlated gene sets were enriched for fatty acid metabolism, TGF-B-related, and other pathways.

**Discussion:** This study identifies the timeline of transcriptional changes in muscle after RC repair, which by itself, does not induce a growth/regenerative response as desired. Instead, it is predominately related to metabolism/energetics changes at 1 week post-repair, unclear or asynchronous transcriptional diversity at 2 weeks post-repair, increased adipogenesis at 4 weeks post-repair, and a low transcriptional steady state or a dysregulated stress response at 8 weeks post-repair.

## 1 Introduction

Rotator cuff (RC) tears are prevalent in the general population over the age of 60 (MacDermid et al., 2004; Yamamoto et al., 2010; Sayampanathan and Andrew, 2017; Collin et al., 2019) with over 400,000 surgical repairs performed in the United States yearly (McElvany et al., 2015). Clinical studies demonstrate that surgical repair does not improve or reverse the muscle atrophy and fatty infiltration developed at chronic states of disease (Gerber et al., 2000; Galatz et al., 2004; Gladstone et al., 2007; Cho and Rhee, 2009; Park et al., 2015). This is counter to what is expected because with muscle loading, as it is well documented that hypertrophy and growth/regeneration transcriptional programs are active (Perry & Rudnick, 2000; Bodine et al., 2001; Flück et al., 2005; Glass, 2005; Dalbo et al., 2011; Egerman & Glass, 2014). This disconnect between reloading and a positive anabolic response could be related to altered mechanotransduction, signaling, transcriptional activity, protein synthesis, or myofibrillar assembly.

The biological activity (specifically transcriptional activity and histology) of muscle after RC tear has been more aggressively investigated than after surgical RC repair in a range of animal models from mice, rats, rabbits, and sheep (Gibbons et al., 2018; Hu et al., 2019; Chaudhury et al., 2016; Choo et al., 2014; Flück et al., 2017 & 2020; Tashjian et al., 2020; Lee et al., 2018; Derwin et al., 2010; Rowshan et al., 2010; Vasquez-Bolanos et al., 2021). All models have advantages and disadvantages. For example, mice and rats require a tenotomy and neurotomy to reproduce the muscle structural phenotypes observed in humans, thereby negating the possibility of a functional repair. Rabbit and sheep appear to undergo fatty infiltration and atrophy changes without the associated nerve injury required in small animals, but sheep are generally more expensive and time intensive, making rabbits an appealing model choice for RC injury and repair.

In the RC surgical repair literature, there is a larger focus on acute repair, where a tenotomy and repair are performed simultaneously, and surgical techniques/biologic augmentation are explored in parallel to acute tendon healing (Ozbaydar et al., 2008; Chung et al., 2013; Honda et al., 2017; Ruoss et al., 2018a; Kwon et al., 2018; Li et al., 2018; Su et al., 2018; Yoon et al., 2018; Sun et al., 2020). However, this does not represent the complex human scenario of delayed repair. For example, in older patients, symptomatic tears develop over years and do not undergo surgical repair for months or years after diagnosis. As a result, there is less literature exploring the more human, and pathophysiologically relevant, approach of performing a delayed repair. When delayed repair is implemented, the model systems are typically sheep and rabbit (Gerber et al., 1999; Gerber et al., 2000; Davidson and Rivenburgh, 2000; Matsumoto et al., 2002; Coleman et al., 2003; Gerber et al., 2004; Rubino et al., 2008; Gerber et al., 2009; Steinbacher et al., 2010; Farshad et al., 2011; Uhthoff et al., 2014; Ren et al., 2018; Ruoss et al., 2018b, & Wu et al., 2022). The rabbit delayed RC repair exhibits an advantage over the sheep model in terms of decreased cost and time.

Similar models, such as disuse followed by resistance exercise, have demonstrated a hypertrophy biological activity response with the reloading of an atrophied muscle (Flück et al., 2005; Dalbo et al., 2011). A delayed repair, likewise, should induce an anabolic response in the muscle, due to reloading of the muscle mechanically. However, to date, there are no time series, transcriptional analyses in muscle after repair in either a sheep or rabbit model system. As a result, it is unclear whether a delayed RC surgical repair promotes growth or regeneration of an atrophied muscle after tear as expected from the loading or reloading of muscle in other model systems. Given the lack of knowledge related to the influence of delayed surgical tendon repair on muscle recovery, there is a need for a pre-clinical model to explore the biological state of the muscle after a tear injury and surgical repair of the tendon. This study aims to fill that gap by elucidating the time-dependent transcriptional changes in muscle after tendon repair using a well-established, chronic rabbit RC tear and repair model. Thus, the primary contribution of this manuscript is a first transcriptome-wide, and time resolved, data set of biological activity after chronic tear and repair. Given the typical anabolic response observed in muscle (re) loading, we hypothesize that a hypertrophy growth and regenerative muscle response will be present at the transcriptional level after reloading of the muscle via surgical repair of the tendon.

## 2 Materials and methods

### 2.1 Animals

In this study 21 skeletally mature female New Zealand White rabbits (∼6 months, Western Oregon Rabbit Company, Philomath, OR) were used to evaluate post-repair transcriptional changes over time. Females were used due to housing safety concerns regarding mixing gender and the ease of sourcing older female animals. All protocols were approved by the University of California, San Diego Institutional Animal Care and Use Committee (protocol #S11246). All animals were assigned a number ID and individual cage location upon arrival and then at time of harvest were randomized to one of the study groups. Animals were single housed with food and water *ad lib*, environmental and food enrichment, and visual access to other animals. There were initially four rabbits in the 1 and 8 weeks post-repair groups, and five rabbits in the 2 and 4 weeks post-repair groups. One rabbit in the 4 weeks post-repair group was sacrificed before repair due to lack of appetite and weight loss, leaving *n* = 4 at every timepoint except 2 weeks post-repair (*n* = 5). These tissues were also used in a prior study of histology (Wu et al., 2022).

### 2.2 Surgical procedures

Rabbits were anesthetized with a subcutaneous injection of ketamine and xylazine (35 mg/kg ketamine/5 mg/kg xylazine, MWI Veterinary Supply, Boise, ID). Following intubation, 2%–4% isoflurane (VetOne, Boise, ID) was utilized to keep the animals under anesthesia for the duration of the surgery. The left supraspinatus muscle served as the experimental side in all animals, with the right shoulder as an unoperated control, as described previously (Vargas-Vila et al., 2021). In brief, an open anterior approach was performed on the left shoulder, followed by sharp transection of the left supraspinatus tendon from its footprint on the greater tuberosity of the humerus. The surrounding soft tissues were bluntly dissected to allow unhindered retraction of the tendon stump and distal muscle. After securing a Penrose drain to the tendon stump to prevent scar formation between the tendon and surrounding soft tissue, the incision was closed in layers. Rabbits were then allowed individual cage activity with routine post-operative care. A fentanyl patch was placed on the back for pain control for 3 days, and the animals were monitored daily for 2 weeks post-operatively. At 8 weeks post-tenotomy, all animals underwent an open repair of the torn tendon. The repair was performed using a modified locking suture with anterior and posterior bone tunnels to restore the tendon footprint to the humeral head. The same anesthesia (subcutaneous injection of ketamine and xylazine), surgical approach and closure, and post-operative protocols (fentanyl patch and daily monitoring) were used for the surgical repair operations (Wu et al., 2022).

Rabbits were monitored two times/day for the duration of the study, once by animal care staff and once by lab staff. In addition to having a fentanyl patch provided at the time of the procedure, animals were monitored for breakthrough pain and distress by looking for signs of chewing or excessive attention to surgical site, reduced food and water consumption, poor self-manicure (lack of grooming or rough coat), abnormal gait, reduced activity or inactivity, teeth grinding, weight loss, vocalizations, and redness and swelling around the eyes. When signs of pain were present, buprenorphine was administered, and a veterinary consult was requested. Of the 16 rabbits, only two rabbits exhibited some swelling, and no additional medication was needed. Quality of the repair was assessed by visual inspection (suture knots are confirmed to be intact and the distal tendon remains approximated to the humerus), and any re-tears or unusual findings were recorded and reported. Only one rabbit had an unusual small region of scar tissue in the muscle.

### 2.3 Muscle harvesting

After the study, animals were euthanized at four time points; 1 week, 2 weeks, 4 weeks, and 8 weeks post-repair. At the specified time points, animals were euthanized with an intravenous overdose of pentobarbital (Beuthanasia, 120 mg/kg, MWI Veterinary Supply, Boise, ID). The supraspinatus muscles from both shoulders were harvested and divided into four regions with the central tendon serving as the muscle midline between the anterior and posterior sides of the muscle. These four regions included anterior lateral (A1), posterior lateral (P1), anterior medial (A2), and posterior medial (P2), and one full-muscle thickness fragment was harvested from each location. The harvested muscle regions were pinned to *in vivo* length and flash frozen in liquid nitrogen-chilled isopentane for storage at −80 C degrees (Wu et al., 2022).

### 2.4 RNA extraction

As previously described (Vasquez-Bolanos et al., 2021) the muscle samples from the P1 region were removed from −80°C and brought to a cryostat where they were allowed to come up to −20°C. The P1 region was chosen due to consistently presenting the most affected region of muscle in this rotator cuff injury model compared to the anterior and medial regions (Vargas-Vila et al., 2021). A 50–75 mg piece was removed from the center of each pinned region and placed in a pyrogen-free tube. RNA extraction was performed using the QIAGEN Fibrous Tissue mini kit on a QIAGEN Qiacube robot (QIAGEN, Germantown, MD). In brief, the tissue was immersed in buffer RLT and disrupted by bead in the QIAGEN TissueLyser II, (QIAGEN, Germantown, MD) before being transferred to the Qiacube for RNA extraction. Samples were digested with Proteinase K, (QIAGEN, Germantown, MD) prior to extraction. A DNase digestion step was included in the protocol. RNA was stored at −80°C.

### 2.5 RNA sequencing

Total RNA was assessed for quality using an Agilent Tapestation 4200, and samples with an RNA Integrity Number (RIN) greater than 8.0 were used to generate RNA sequencing libraries using the TruSeq Stranded mRNA Sample Prep Kit (Illumina, San Diego, CA). Samples were processed following manufacturer’s instructions, modifying RNA shear time to 5 minutes. Resulting libraries were multiplexed and sequenced with 75 basepair (bp) single reads (SR75) to a depth of approximately 25 million reads per sample on an Illumina HiSeq400. Samples were demultiplexed using bcl2fastq Conversion Software (Illumina, San Diego, CA).

### 2.6 RNAseq analysis

Quality control of the raw fastq files was performed using the software tool FastQC (Andrews, 2010). There was one animal that was partially filtered out at this step, the experimental sample at the 8 weeks time point. Sequencing reads were aligned to the rabbit genome (Ensembl OryCun2.0) using the STAR v2.5.1a aligner (Dobin et al., 2013). Read quantification was performed with RSEM (Li and Dewey, 2011) (v1.3.0) and Ensembl annotation (Oryctolagus_cuniculus.OryCun2.0.91.gtf). The R BioConductor packages edgeR (Robinson et al., 2010) and limma (Ritchie et al., 2015) were used to implement limma-voom (Law et al., 2014) followed by empirical Bayes technique for differential expression analysis. Lowly expressed genes were filtered out (cpm >1 in at least one sample). Trimmed mean of M-values (TMM) normalization was applied (Robinson and Oshlack, 2010). The experimental design was modeled upon time point and treatment (∼0+ time_treatment) with contrasts (Repair–Control) for each time point and all samples. All results will be presented as the tenotomy time point compared to sham unless specified otherwise. From the empirical Bayes result, differentially expressed (DE) genes were defined by an adjusted *p*-value <0.05 [based on the moderated t-statistic using the Benjamini–Hochberg (BH) method to control the false discovery rate (Benjamini et al., 2001)] and a |log2FC| >1 (Supplementary Data S1).

To identify human orthologs of rabbit genes, G:Profiler was used to map rabbit Ensemble IDs to human Ensemble IDs, Entrez IDs and symbols (Raudvere et al., 2019) (Supplementary Data S2). Of the 11,604 total genes, 1,237 were not mapped to human and 255 were duplicates and were removed for the analysis. The resulting genes with Entrez IDs correspond to the set of “background or detected genes” consisting of 10,112 genes.

Based on the biological coefficient of variation of 0.419 observed in our data, with a sequencing depth of 20 million reads/sample, we are powered to detect a 2-fold change with 64% power with *n* = 4 per group. Volcano plots were created using Enhanced Volcano package (v.1.60). Heatmaps were created using clustermap in the seasborn package (Waskom, 2021). The Venn Diagram was produced using Van de Peer lab tools. RT-qPCR validation was not used in this study due to the robust nature of RNAseq methods and data analysis and supporting literature (Coenye, 2021; Feng et al., 2010).

### 2.7 Enrichment analysis

Assignment of functional categories was based on the Gene Ontology (GO) categories “Biological process,” “Molecular function,” and “Cellular component.” Enrichment analysis of GO categories was performed in R (version 4.0.2; http://www.r-project.org) using the “weight01” method from the Bioconductor topGO (v. 2.40.0) package with the org.Hs.eg.db_3.11.4 human database (Carlson, 2019; Alexa & Rahnenfuhrer, 2020). Node size was set to 10, and Fisher’s exact test was used for assessing GO term significance. Overrepresentation of functional categories was calculated for DE genes as compared with the 10,112 “background” genes, and significant GO terms were identified as those having *p*-value <0.05 (Supplementary Data S3). KEGG pathway analysis was also done in R using KEGGREST package (v. 1.28.0) with list of pathways and genes. A Wilcox rank-sum test was performed for each pathway, where Entrez ID along with the adjusted *p*-values results were used as inputs. Overrepresentation of KEGG pathways was calculated for DE genes as compared with the 10,112 “background” genes, and significant KEGG pathways were identified as those having a *p*-value <0.05 (Supplementary Data S4). All pathways with at least one time point with a significant *p*-value and filtering out disease/tissue specific pathways, the remaining pathways were grouped by KEGG hierarchy into amino acid metabolism, carbohydrate metabolism, vitamins and cofactors metabolism, energy metabolism, lipid metabolism, endocrine system, nervous system, immune system, signal transduction, cell processes.

### 2.8 Biased gene pathways analysis

To explore well known genes involved in muscle homeostasis and pathology, we specifically probed myogenesis, anti-myogenesis, inflammation, adipogenesis, and fibrosis programs with genes defined by the literature (Gibbons et al., 2018; Shahidi et al., 2020; Vasquez-Bolanos et al., 2021).

### 2.9 Correlation analysis

Weighted correlation network analysis (WGCNA) was performed using WGCNA R package (v. 1.70–3) (Langdelder and Horvath, 2008) with transcriptional and phenotypic data (Supplementary Data S5) with the repair only samples in order to correlate histological traits with changes due to repair. This analysis identified unbiased modules or sets of genes that are clusters of highly correlated genes (Supplementary Data S8). In the combined analysis (both repair and control groups) only two modules correlated significantly with fat. This analysis works by building an unbiased network of modules which represents a cluster of genes, and then correlations (Supplementary Data S6) can be investigated with phenotype traits through gene membership. GO enrichment analysis was then performed by GoEnrichmentAnalysis function within the WGCNA R package, and returns the top 10 GO terms of each module (Supplementary Data S7). The phenotypic data included in this study is fiber area, central nucleation, fat quantification, collagen content, and degeneration, all of which are reported in detail elsewhere (Wu et al., 2022) but are from the same animals used in this study.

## 3 Results

### 3.1 Transcriptome profile changes

**FIGURE 1:**
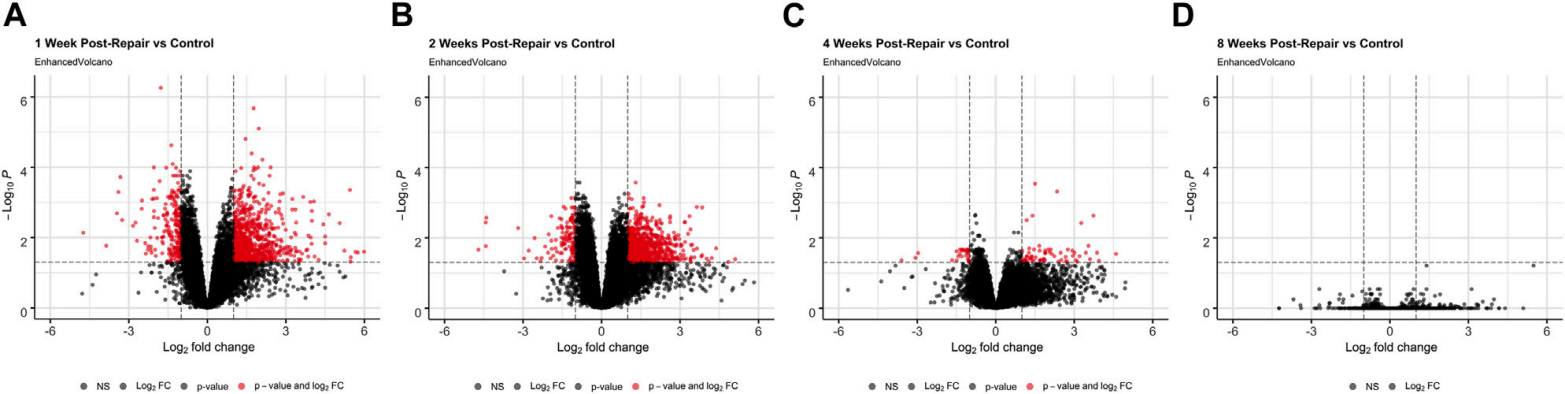
Volcano plots **(A–D)** highlight differentially expressed (DE) genes (red dots) at each time point post-repair defined as a |logFC| >1 and an adjusted *p*-value <0.05 [based on the moderated t-statistic using the Benjamini–Hochberg (BH)].

**FIGURE 2:**
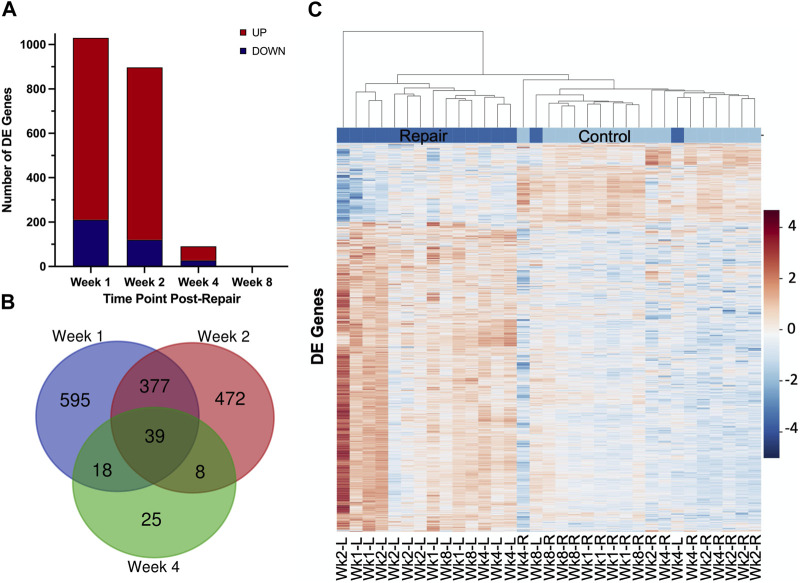
Distribution of samples by repair vs. control and DE genes over each time point post-repair. Venn diagram **(A)** highlights the DE genes at each time point and the overlap with other time points. The bar chart **(B)** displays the number of DE genes which are up or downregulated at each timepoint. Data in the heatmap **(C)** is presented as normalized expression for each repair (L = left) and control (R = right, unoperated shoulder) sample at each time point post-re with a z-score scale by rows and an average hierarchical clustering by columns.

At 1 week post-repair compared to control, about 80% of the DE genes were upregulated (Figures 1A, 2A) with a similar trend at 2 weeks (Figures 1B, 2A), and 10% of the total number of DE genes at 4 weeks post-repair (Figures 1C, 2A). Meanwhile, at 8 weeks post-repair compared to controls, there were no DE genes (Figures 1D, 2A). The total amount of significant DE genes by week was 1,029 (819 up and 210 down) at 1 week, 896 (776 up and 120 down) at 2 weeks, 90 (63 up and 27 down) at 4 weeks, and 0 at 8 weeks post-repair (Supplementary Data S1). Comparing DE genes between time points, there are 595 unique DE genes at 1 week post-repair, 472 at 2 weeks, 25 at 4 weeks, and none at 8 weeks post-repair. There are 39 genes in common at all the time points that have DE genes (Figure 2B). Week 1 and week 2 uniquely share 377 genes, week 1 and 4 share 18 genes, and week 2 and 4 share 8 genes (Figure 2B). Post-repair gene expression was associated with more upregulation compared to control (Figure 2C). Specifically, post-repair times points 2 and 4 weeks cluster strongly together and weeks 1 and 8 also cluster together, while the control weeks 1, 2, and 4 cluster strongly with 8 weeks (Figure 2C).

### 3.2 Enrichment analysis

**FIGURE 3:**
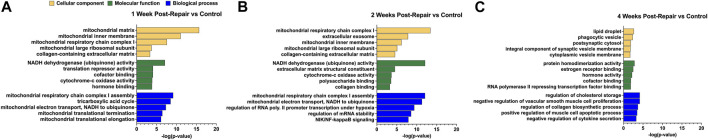
GO enrichment analysis for **(A)** 1 week post-repair, **(B)** 2 weeks post-repair, and **(C)** 4 weeks post-repair. Data presented as top 5 most significant term in each category: cellular component (yellow), molecular function (green), biological process (blue) (Supplementary Data S3).

**FIGURE 4:**
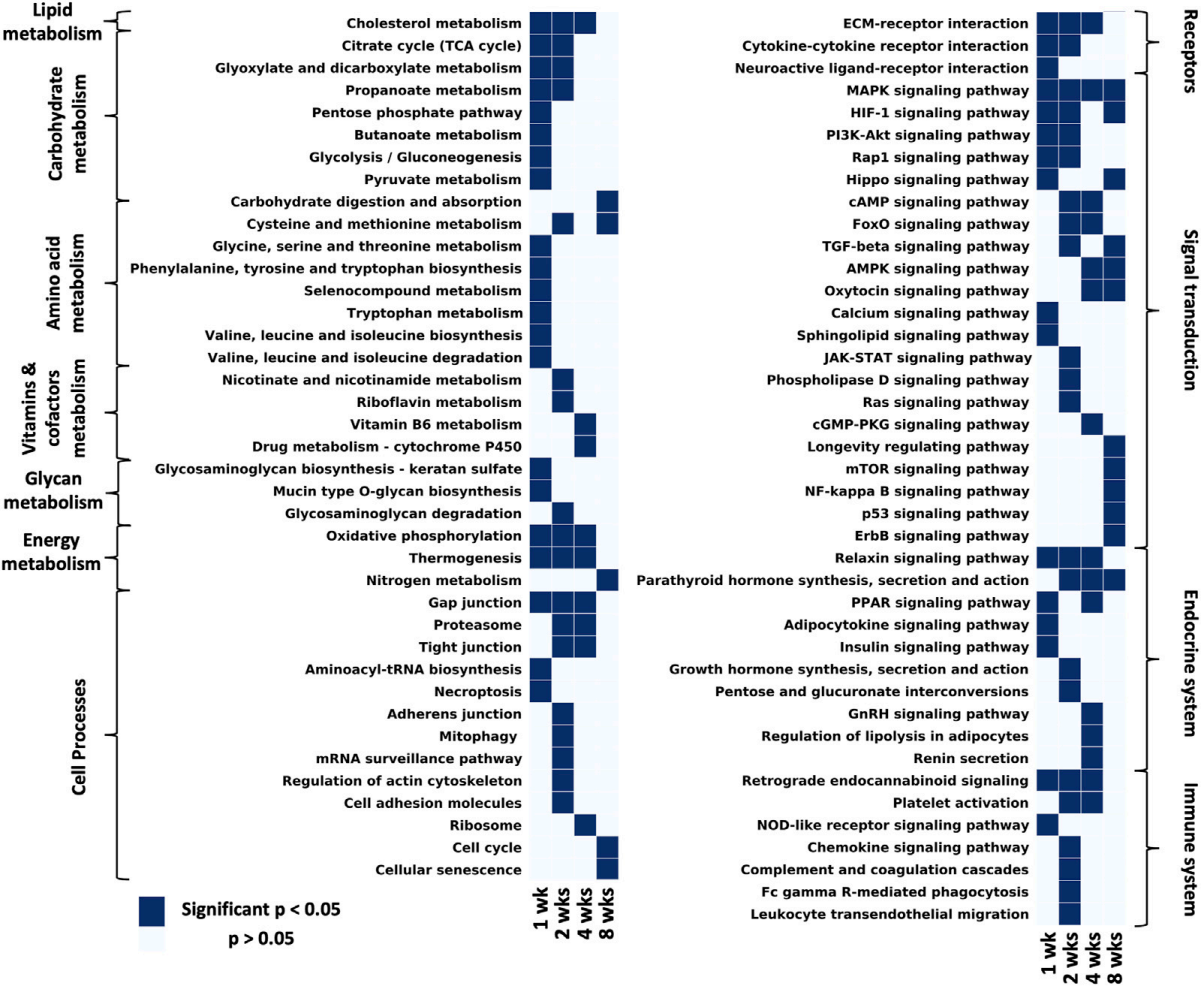
Considering all pathways that have at least one time point with a significant *p*-value and filtering out disease/tissue specific pathways, the remaining pathways were grouped by KEGG hierarchy into amino acid metabolism, carbohydrate metabolism, vitamins and cofactors metabolism, energy metabolism, lipid metabolism, endocrine system, nervous system, immune system, signal transduction, cell processes (Supplementary Data S4).

To determine the biological relevance of the DE genes, overrepresentation analyses were performed using GO and KEGG at each time point (Figures 3, 4, Supplementary Datas S3, S4). For 1 week post-repair, GO cellular component terms (Figure 3A) were enriched for parts relating to the mitochondria and collagen ECM (*p*-value 0.00046). GO molecular function terms (Figure 3A) were enriched for ubiquinone (*p*-value 0.000000085) and cytochrome c activity (*p*-value 0.00013) along with translation repressor activity and cofactor and hormone binding (*p*-value 0.000082; 0.000084, 0.00015). GO Biological process terms (Figure 3A) were enriched for the citric acid (TCA) cycle, mitochondrial electron transport chain components, and mitochondrial translational elongation and termination (*p*-value 0.0000000031; 0.000000047; 0.00000076; 0.00000036).

GO analysis at 2 weeks post-repair terms were enriched for components of the mitochondria and ECM, in addition to extracellular exosome (*p*-value 0.000000000000036; 0.000027; 0.000000015). For molecular function, DE genes were enriched for pathways of ubiquinone and cytochrome-c activity (*p*-value 0.00000000000079; 0.00021) along with polysaccharide and collagen binding (*p*-value 0.00027; 0.00054). The electron transport chain activity continues in the biological process terms along with regulation of transcription in response to hypoxia, regulation of mRNA stability, and NIK/NF-kappaB signaling (*p*-value 0.0000000000008; 0.0000000004; 0.0000000025; 0.000000012; Figure 3B).

At 4 weeks post-repair the DE genes are no longer enriched for cellular components terms of mitochondrial components and instead for lipid droplet, phagocytic vesicle, postsynaptic cytosol, synaptic vesicle membrane, and cytoplasmic vesicle membrane (*p*-value 0.0022; 0.0043; 0.0134; 0.0162; 0.0191; Figure 3C). Molecular function at 4 weeks had similar DE gene enrichment for hormone and cofactor binding (*p*-value 0.0065; 0.0126) as 1 week post-repair, in addition to RNA polymerase II repressing transcription factor binding, estrogen receptor binding, and protein homodimerization activity (*p*-value 0.0141; 0.0032; 0.0015; Figure 3C). DE genes were enriched for regulation of a range of processes dominates at 4 weeks in biological process including regulation of cholesterol storage, collagen biosynthetic process, and negative regulation of vascular smooth muscle cell proliferation, and cytokine secretion, and positive regulation of muscle cell apoptotic process (*p*-value 0.000067; 0.0002; 0.000067; 0.00052; 0.00031; Figure 3C).

KEGG analyses highlight different pathways that were enriched at each time point (significantly enriched means *p*-value <0.05, exact *p*-values can be found in Supplementary Data S4). At 1 week post-repair most terms in lipid, carbohydrate, amino acid, glycan and energy metabolism were significantly enriched. However by 2 weeks there is over a 50% reduction of significantly enriched metabolism terms with even fewer enriched at 4 and 8 weeks post-repair (Figure 4 metabolism). One category to note of metabolism is energy metabolism, which was uniquely enriched from 1 to 4 weeks post-repair. Cell processes were mainly enriched over time for gap junctions and the proteasome, with the majority of terms enriched at 2 weeks including: mitophagy, mRNA surveillance pathway, regulation of actin cytoskeleton, cell adhesion, and adherens junction (Figures 4 Cell processes). Receptor interaction for the ECM was enriched from 1 to 4 weeks post-repair, with cytokine and neuroactive ligand interactions enriched at few earlier time points. In the signal transduction category, MAPK signaling was enriched at all time points with the number of enriched pathways increasing at each time point. At 8 weeks post-repair, there were five uniquely enriched signaling pathways: longevity regulating, mTOR, NF-kappaB, p53, and ErbB (Figure 4 signaling transduction). The endocrine system terms were most enriched at 4 weeks post-repair where in particular, regulation of lipolysis in adipocytes was uniquely enriched (Figure 4 endocrine system). Meanwhile, at 2 weeks the most immune system terms were enriched and uniquely: chemokine signaling, leukocyte migration, phagocytosis, complement and coagulation cascades (Figure 4 immune system).

### 3.3 Gene programs of interest changes over time after repair—Muscle specific

**FIGURE 5:**
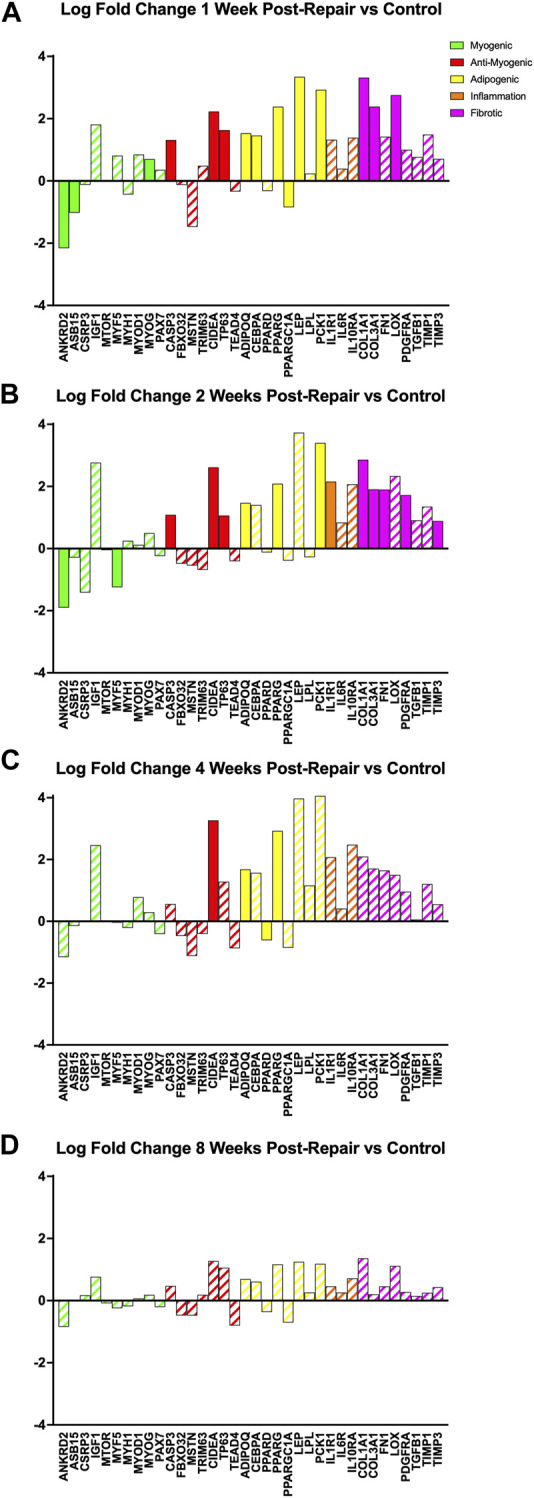
Changes in transcriptome of genetic programs of interest at **(A)** 1 week post-repair, **(B)** 2 weeks post-repair, **(C)** 4 weeks post-repair, and **(D)** 8 weeks post-repair. Log fold change (logFC) is the difference of repair and control (Supplementary Data S5). Solid bars represent a significant adjusted *p*-value (*p* < 0.05) and partially filled (hatched) in bars are not significant. Green bars represent myogenic related genes, red represents anti-myogenic, yellow represents adiopogenic, orange represents inflammation and pink represents fibrotic genes. Data are presented as average logFC.

All programs, relating to myogenesis, anti-myogenic (suppressing muscle formation, cell death, degradation), adipogenesis, and fibrosis, had a few significant DE genes (|logFC| >1 & adj. *p*-value <0.05) at 1 week post-repair (Figure 5A). At 2 weeks post-repair, there were even fewer significant DE genes for the myogenic and adipogenic categories, and more significant DE genes for the inflammation and fibrotic programs (Figure 5B). In fact, at 4 weeks post-repair there were only significant DE genes in the anti-myogenic and adipogenic programs (Figure 5C). There were no significant DE genes at 8 weeks post-repair and the corresponding magnitude of expression is also much lower compared to all other time points (Figure 5D).

### 3.4 Transcriptional data correlations with phenotypic traits

**FIGURE 6:**
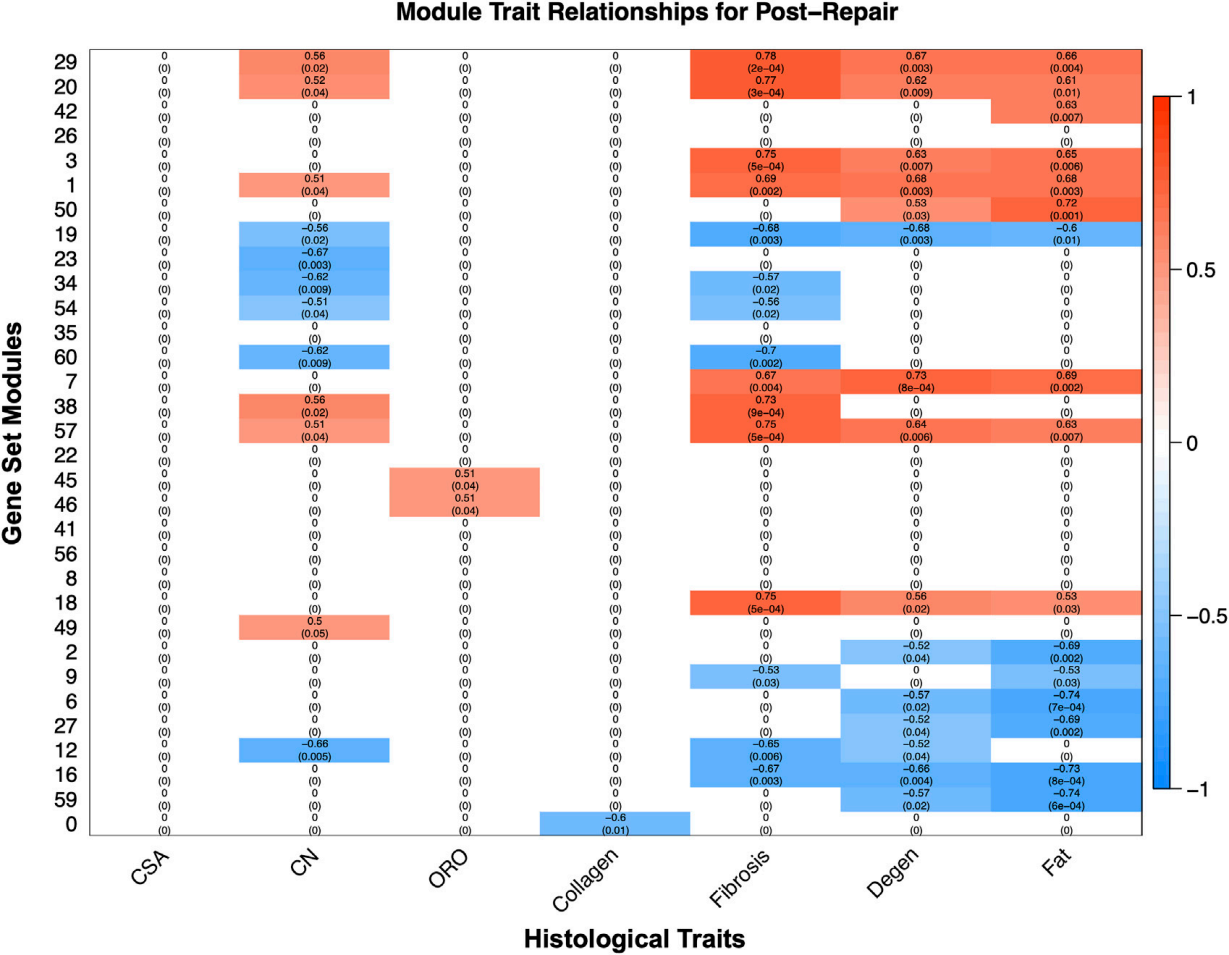
The modules, unbiased clustering of genes (described in Supplementary Data S8), on the left and the phenotypic traits are on the bottom. The scale bar represents the correlation coefficient and is the first number listed in each cell, and the number in parentheses is the *p*-value. Only cells with a *p*-value equal or less than 0.1 were selected to be displayed and the white cells with zeros represent cells with a *p*-value >0.1 for clarity of which modules correlate with which phenotypic traits (Supplementary Data S6).

WGCNA revealed gene modules significantly related to phenotype characteristics quantified by histology (Figure 6). Transcriptional activity correlated significantly with histological traits such as fat, degeneration, fibrosis, and centralized nuclei (CN), oil red-O (ORO) and collagen (Figure 6). The most positively correlated module gene set for degeneration (module 7 corr 0.731864336; *p*-value 0.000770984) was enriched for TGF-B related pathways, and for fat (module 50 corr 0.722057657; *p*-value 0.001008972) was enriched for fatty acid metabolism, predominately catabolism (Figure 6). Module 29 was positively correlated for CN (corr 0.562698044; *p*-value 0.021680671), fibrosis (corr 0.781338353; *p*-value 0.000155891), degeneration (corr 0.673602659; *p*-value 0.003210287), and fat (corr 0.658490829; *p*-value 0.004386982), and was enriched for vesicular transport and phosphatase binding (Figure 6, Supplementary Data S7).

## 4 Discussion

The purpose of this study was to establish transcriptional changes as a function of time in a pre-clinical RC surgical repair model. We hypothesized, based on the typical behavior of reloading muscle, we would observe a hypertrophy growth and regenerative muscle response after surgical repair. However, the lack of a transcriptional regenerative/growth response, at any time point, and the lack of positive morphological changes measured in this model (Wu et al., 2022), suggest that surgical repair of the tendon after a chronic tear does not induce the expected anabolic responses in the muscle. Transcriptionally, it appears that, due to the previous tear injury, there is a reduced initial wound healing response and a quicker transition towards inflammation, fatty infiltration, apoptosis, and fibrosis compared to the tear alone model (Vasquez-Bolanos et al., 2021). Likewise, phenotypic traits correlated significantly with gene groupings in unbiasedly defined modules which were enriched for biological relevant pathways such as fatty acid metabolism and in particular catabolism.

After surgical repair there was a strong initial response of over 1,000 differentially expressed (DE) genes which decreased to zero by 8 weeks post-repair where across each time point the majority of DE genes were upregulated (Figures 1A–D; Figure 3B). Of the time points with DE genes, there were more unique genes than genes shared between time points (Figure 2A), highlighting that there were shifts in biological programs of the DE genes in the muscle at each time point. Broadly, functional enrichment determines that 1-week post-repair was primarily related to metabolism and energetic activity, cofactor/hormone binding, and mitochondrial translation (Figures 3, 5). Meanwhile, for muscle specific genes there are various significant DE genes in each category except inflammation (Figure 5). This differs from the 1 week post-tenotomy transcriptional response in a rabbit model, mouse model (1 week) and a rat tenotomy and denervation (10 days), where inflammatory genes were overexpressed and there was far less significant adipogenic genes (Vasquez-Bolanos et al., 2021; Lee et al., 2018; Gumucio et al., 2019). Similar enriched pathways to 1 week post-repair were present at 2 weeks post-repair, along with NIF/NF-kappaB signaling, transcription in response to hypoxia, and mRNA stability, making this time point the most transcriptionally diverse (Figures 3–5). Histologically, the most muscle atrophy was observed at this time point (Wu et al., 2022), although transcriptionally it is not obvious as to why that may be. Many KEGG terms are enriched across the board including cell processes such as mitophagy, and signal transduction such as JAK-STAT, phospholipase D, Ras, and TGF-B signaling pathways (Figure 4). In particular, the immune system response is most pronounced at 2 weeks post-repair time point (Figure 4) and unlike the response observed in previously mentioned rabbit (1, 2, 4, 8, and 16 weeks) mouse (1 and 4 weeks) and rat (10, 30, 60 days) RC tear models investigating time-dependent transcriptional response, where the strongest immune system was at the first recorded time point, and decreased from there (Lee et al., 2018; Gumucio et al., 2019; Vasquez-Bolanos et al., 2021). This increase in inflammation related transcription may indicate the chronic inflammatory state in which repair is performed and the possibility of further activating the immune system processes which continues to dysregulate the cellular environment. Additionally, there are enriched programs involved in mitochondrial energetics and the ECM, particularly related to collagen, where extracellular space and calcium binding was also enriched in a sheep RC tear model (Flück et al., 2017; Flück et al., 2020). Overall, 2 weeks post-repair was active for inflammation, cellular environment, and cellular homeostasis (likely apoptosis) programs with the beginning signs of fatty acid activity. This highlights the difference between RC tear transcriptional data across a range of animal models, adipogenic program terms are enriched as early as 1 week post-repair in comparison to about a month post-tear in other studies (Lee et al., 2018; Gumucio et al., 2019; Hu et al., 2019; Vasquez-Bolanos et al., 2021). This is unsurprising when comparing to torn human RC muscle, because of the characterization for high-fat (∼40%) muscle (Gibbons et al., 2018), and animal models struggling to recapitulate a representative percentage of fat (i.e., Rabbit ∼10–15% fat), so seeing the transcriptional presence of adipogenesis supports the rabbit chronic tear and repair model’s similarity to human.

The strongest shift towards lipids, fibrosis, hormones, apoptosis, and cytokine activity occurred at 4 weeks post-repair even with an overall decrease in the number of differentially expressed genes (Figures 3, 5). At 8 weeks post-repair, there were no DE genes (Figures 1, 3), possibly representing a steady state of gene expression. This decrease of expression and DE genes was also observed in a post-tenotomy model possibly representing a steady state reached after an invasive event such as tenotomy or repair (Vasquez-Bolanos et al., 2021). However, when considering significant genes that did not meet the log fold change minimum, KEGG enrichment could be assessed and terms related to fibrosis, energetics, cell fate, inflammation, highlighting general dysregulation with stress response (Figure 4).

After describing the transcriptional profile at each time point post-repair, the key finding is that there is no growth/regenerative signal as one would expect from muscle (re)loading. Instead, there are energetic changes, inflammation, lipid metabolism, phagocytosis, and apoptosis, accentuating the reality that tendon repair does not induce the desired fundamental biological result. In addition, histological changes correlated with transcriptional results, although these changes were more general in observation, the transcriptional data provides the opportunity to sparse apart which programs may be active and when. However, the possibility of whether this state is permanent or reversible becomes an increasingly interesting future direction. Further validating studies to investigate in the future include; measuring muscle cell signaling responses to reloading, identifying the cell types present and activated by repair using immunohistochemistry or immunofluorescence, and exploring how the immune system interacts with muscle in this state of dysfunction. Similarly, using proteomic approaches to further validate transcriptional data will be important.

Limitations of this study include; the inability to determine direct cause-effect relationships with gene sets of interest, the need to validate the observed transcriptional profile with proteomic data, and the lack of comparative literature quantifying transcriptional data post-repair. This limits the ability to make specific conclusions and compare to well defined baselines or expected changes to reloading after chronic injury. Another limitation is the sample size and power of this study. In order to achieve a power of >80% with this sequencing depth, one would need a sample size of 7. As such, false negative rates could be higher than desired. However, the genes of interest (growth and regeneration) are clearly not differentially regulated and do not demonstrate variance structures that indicate lack of power in our primary hypothesis. Using only female rabbits is also a potential limitation as it relates to extrapolating to males. This could not be avoided because we use animals that have reached skeletal maturity and sourcing is only available in females of this age. Likewise, the age of this rabbit model (∼10 months at the end of the experiment) is not a direct analog to aging-related muscle changes that would be expected with the older human population experiencing a rotator cuff tear and delayed repair. Lastly, the amount of time that is allowed to pass between tenotomy and surgical repair could influence the findings (Uhthoff et al., 2014), this study aimed to use a duration (8 weeks) where characteristic pathophysiology is present and additional changes to the muscle are minimal.

## 5 Conclusion

Defining transcriptional changes in a preclinical RC surgical repair model such as rabbit allows for the possibility of further mechanistic studies to understand the role surgical repair plays on the muscle dysfunction leading to continued muscle atrophy and fatty infiltration observed. These data will also provide a scientific premise for future investigations aimed at understanding the effect of repair plus adjuvant therapeutics, and help tease apart the potential impact of adjuvant therapies versus surgical repair techniques. This study identifies the timeline of transcriptional changes in muscle after RC repair, which by itself, does not induce a growth or regenerative response as desired and instead is predominately related to metabolism/energetics changes at 1 week post-repair, unclear or asynchronous transcriptional diversity at 2 weeks post-repair, increased adipogenesis at 4 weeks post-repair, and a low transcriptional steady state or a dysregulated stress response at 8 weeks post-repair. Given the lack of a positive regenerative/growth response in the presence of mechanical reloading, future experiments are being directed at mechanical connectivity within the muscle, mechanical signaling processes, and epigenetic changes in myocytes that may individually (or collectively) preclude a positive muscle response to reloading.

## Data Availability

The datasets presented in this study can be found in online repositories. The names of the repository/repositories and accession number(s) can be found below: https://www.ncbi.nlm.nih.gov/geo/, GSE186320.
