# Supplementary material for: Transcriptional time course after rotator cuff repair in 6 month old female rabbits
Source: Front Physiol. 2023 May 9;14:1164055. doi: 10.3389/fphys.2023.1164055 (PMC10203179; doi:10.3389/fphys.2023.1164055)
Supplement: Supplementary file 2 [file Table1.DOCX]

Supplementary Material

# Supplementary Data

Data 1. DE matrix where a 1 represents an up-regulated differentially expressed gene and a -1 represents a down-regulated differentially expressed gene. The DE genes for each time point are included.

Data 2. Toptable includes the mapping from rabbit Ensembl to human Ensembl “GENEID”, ENTREZID, and SYMBOL for all genes recorded. As well as, for each time point and each gene, it includes the logFC (log_2_ fold change), AveExpr (average expression across all samples, in log2 CPM), t (logFC divided by its standard error), p-value (raw p-value based on t, from test that log FC differs from 0), adjusted p-value (Benjamini-Hochberg false discovery rate adjusted p-value), and B (log-odds that gene is DE) .

Data 3. GO terms for biological process (BP), molecular function (MF) and cellular component (CC) for each time point includes GO ID, name of term, number of genes in term, number significant, number of genes expected, and p-value. Yellow highlight represents the terms included in Figure 3.

Data 4. All KEGG results includes the pathway code, name, p-value, number of genes annotated in pathway, and total genes related to pathway. Then all significant pathways (p<0.05) for at least one time point were separated and then filtered to remove disease and less relevant terms. The last two sheets include the final list and grouping used to create heatmap, and at what time points it was significant in Figure 4.

Data 5. Metadata of tenotomy samples and phenotypic characteristics collected used for WGCNA.

Data 6. Full correlation matrix of module number by phenotypic trait, including correlation values (in the first 8 columns) and p-values (in the following 8 columns). Partially displayed in Figure 6.

Data 7. GO enrichment analysis of genes assigned to each module with module size, number of genes found in GO terms as background, the rank of the enrichment (top 10), p-values both adjusted and unadjusted, number of module genes and background, GO term ID, ontology, name and definition.

Data 8. Modules from the repair WGCNA and the corresponding genes and their labels: “GENEID”, ENTREZID, and SYMBOL.
